# Assessing the Novel Mixed Tutton Salts K_2_Mn_0.03_Ni_0.97_(SO_4_)_2_(H_2_O)_6_ and K_2_Mn_0.18_Cu_0.82_(SO_4_)_2_(H_2_O)_6_ for Thermochemical Heat Storage Applications: An Experimental–Theoretical Study

**DOI:** 10.3390/molecules28248058

**Published:** 2023-12-13

**Authors:** João G. de Oliveira Neto, Jailton R. Viana, Antonio D. da S. G. Lima, Jardel B. O. Lopes, Alejandro P. Ayala, Mateus R. Lage, Stanislav R. Stoyanov, Adenilson O. dos Santos, Rossano Lang

**Affiliations:** 1 Center for Social Sciences, Health and Technology, Federal University of Maranhão—UFMA, Imperatriz 65900-410, MA, Brazil; 2 Department of Physics, Federal University of Ceará—UFC, Fortaleza 65455-900, CE, Brazil; 3 Coordination of the Science and Technology Course, Federal University of Maranhão (UFMA), Campus Balsas, MA-140, km 04, Balsas 65800-000, MA, Brazil; 4 Natural Resources Canada, CanmetENERGY Devon, 1 Oil Patch Drive, Devon, AB T9G 1A8, Canada; 5 Institute of Science and Technology, Federal University of São Paulo—UNIFESP, São José dos Campos 12231-280, SP, Brazil

**Keywords:** single-crystal growth, mixed Tutton salts, Hirshfeld fingerprint plots, DFT calculations, thermochemical compounds

## Abstract

In this paper, novel mixed Tutton salts with the chemical formulas K_2_Mn_0.03_Ni_0.97_(SO_4_)_2_(H_2_O)_6_ and K_2_Mn_0.18_Cu_0.82_(SO_4_)_2_(H_2_O)_6_ were synthesized and studied as compounds for thermochemical heat storage potential. The crystallographic structures of single crystals were determined by X-ray diffraction. Additionally, a comprehensive computational study, based on density functional theory (DFT) calculations and Hirshfeld surface analysis, was performed to calculate structural, electronic, and thermodynamic properties of the coordination complexes [M^II^(H_2_O)_6_]^2+^ (M^II^ = Mn, Ni, and Cu), as well as to investigate intermolecular interactions and voids in the framework. The axial compressions relative to octahedral coordination geometry observed in the crystal structures were correlated and elucidated using DFT investigations regarding Jahn–Teller effects arising from complexes with different spin multiplicities. The spatial distributions of the frontier molecular orbital and spin densities, as well as energy gaps, provided further insights into the stability of these complexes. Thermogravimetry, differential thermal analysis, and differential scanning calorimetry techniques were also applied to identify the thermal stability and physicochemical properties of the mixed crystals. Values of dehydration enthalpy and storage energy density per volume were also estimated. The two mixed sulfate hydrates reported here have low dehydration temperatures and high energy densities. Both have promising thermal properties for residential heat storage systems, superior to the Tutton salts previously reported.

## 1. Introduction

In recent years, a diversity of hydrate and anhydrous double salts based on metal sulfates have been synthesized and investigated due to their structural, thermal, and optical properties, promising for use in electrochemical, thermochemical, and optics devices [1,2,3,4,5]. Inorganic crystalline structures, such as langbeinite [6], kröhnkite [7], leonite [8], picromerite [9], blödite [10], alluaudite [11], and vanthoffite [12], are the main classes of materials studied for these purposes because of their composition and structural and chemical stability.

The picromerites belong to a hexahydrate isomorphic crystallographic family called Tutton salts [13]. Depending on the constituents, these double salts can be synthesized in various compositions, imparting diverse and valuable properties [14,15]. Even with such variability in terms of chemical elements and concentrations, picromerites tend to crystallize in a monoclinic symmetry of space group *P*2_1_/*a*, with two formulas per unit cell (Z = 2) [16,17]. These types of double salts have a general chemical formula of M^I^_2_M^II^(XO_4_)_2_(H_2_O)_6_, where M^I^ is a monovalent cation (Cs^+^, K^+^, Rb^+^, Tl^+^, and NH_4_^+^); site M^II^ is occupied by a bivalent cation (Mg^2+^, V^2+^, Mn^2+^, Fe^2+^, Co^2+^, Ni^2+^, Cu^2+^, Zn^2+^, and Cd^2+^); and X are elements in high oxidation states, such as Se and S [18,19]. The M^II^ aquo-cation is hexacoordinated in the unit cell, forming a complex with slightly distorted octahedral geometry [20]. The structural distortion is associated with the well-known Jahn–Teller effect, in which the *d*-level atomic orbitals are split into two sets of degenerate states to reduce the energy of the system [21,22].

The crystal packing of Tutton salts is formed from three molecular moieties, M^I+^, [M^II^(H_2_O)_6_]^2+^, and [XO_4_]^2−^, where the octahedral aqua-complexes form hydrogen bonds with [XO_4_]^2−^-tetrahedrons and weak interactions with M^I+^ [23]. However, these interactions can be distorted by the presence of internal agents, such as impurities, dopants, and the partial occupancy of two or more ions in a single type of site of the host matrix [24,25,26]. The simultaneous introduction of species with different concentrations in the same lattice environment yields a subclass of these double salts, called mixed crystals, in which new properties can be achieved or tuned by the combination of characteristics of the individual moieties [27]. 

Several mixed Tutton salts, such as K_2_Ni_x_Co_1−x_(SO_4_)_2_(H_2_O)_6_ [28], (NH_4_)_2_Ni_x_Co_1−x_(SO_4_)_2_(H_2_O)_6_ [15], (NH_4_)_2_Mn_1−x_Zn_x_(SO_4_)_2_(H_2_O)_6_ [29], K_2_Zn_x_Mg_1−x_(SO_4_)_2_(H_2_O)_6_ [30], and (NH_4_)_2_Ni_x_Cu_1−x_(SO_4_)_2_(H_2_O)_6_ [31], have been explored as materials for ultraviolet light filters and solar-blind devices. Recently, there has been great interest in thermochemical applications, where materials can store energy in the heat form by heating water [2,3,32,33,34]. In this scenario, five criteria have been established for this purpose: (i) dehydration temperature < 120 °C; (ii) energy density ≥ 1.3 GJ/m^3^; (iii) cyclability ≥ 10; (iv) hydration temperature > 50 °C; and (v) hydration vapor pressure < 12 mbar [3,33].

Nevertheless, any application direction primarily involves fundamental science at an experimental and theoretical level. Computational investigations of [M^II^(H_2_O)_6_] hexa-aqua metal complexes can undoubtedly contribute towards a better understanding of the properties of mixed Tutton salt [35]. It is possible to elucidate the structural features of the coordination complexes, as well as the electronic and thermodynamic properties, via electronic structure calculations, which are important for exploring their behavior and possible technological applications [36].

The Jahn–Teller effect, for example, is related to the electronic configuration of the transition metal complex and results in a geometrical distortion that causes modifications in the crystalline structure, such as volume changes and formation energy, which can be studied computationally [37,38]. Density functional theory (DFT) is a powerful tool for developing electronic structure calculations and is particularly effective for clarifying the properties of materials. In this sense, the PBE1PBE functional is considered a practical approach for investigating first-row transition metal complexes [39,40]. Other computational tools that provide valuable insights into the structure of the primitive unit cell and the chemical bonds among molecular moieties are Hirshfeld surfaces and crystal voids analysis [41,42]. Therefore, such computational insights can be effectively applied in combination with experimental results to support a specific material property [43].

Motivated by these exploratory frameworks, in this work, the synthesis of two novel mixed Tutton salts with the chemical compositions of K_2_Mn_0.03_Ni_0.97_(SO_4_)_2_(H_2_O)_6_ and K_2_Mn_0.18_Cu_0.82_(SO_4_)_2_(H_2_O)_6_ was carried out, aiming at thermochemical application. X-ray diffraction (XRD) determined the structures of single crystals. DFT calculations on the [M^II^(H_2_O)_6_] hexacoordinated complexes, where M^II^ = Mn, Ni, and Cu, revealed their key electronic, structural, and thermodynamic properties, as well as information about their contributions to the formation of the materials. Additionally, computational studies, including Hirshfeld surface analysis and crystal voids determination, were conducted to understand the intermolecular interactions in these double salts comprehensively. Finally, the samples were characterized by thermoanalytical techniques to evaluate the possibility of using these mixed Tutton salts in thermochemical heat storage devices.

## 2. Results and Discussions

### 2.1. Crystal Growth

**Figure 1:**
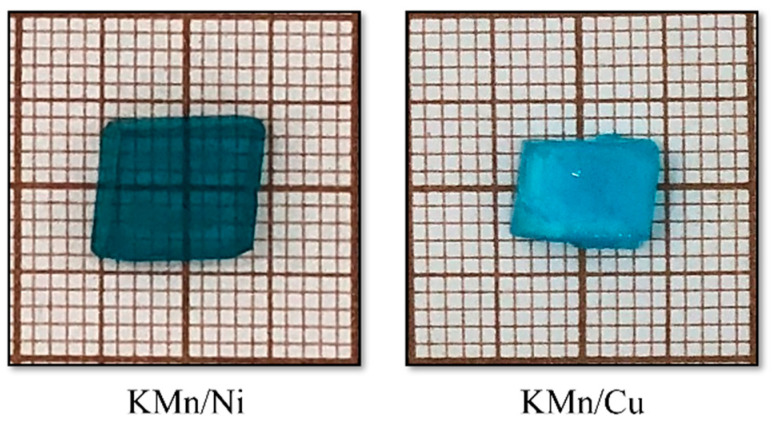
Photographs of as-grown crystals formed by the slow evaporation technique.

The mixed Tutton crystals KMn/Ni and KMn/Cu were successfully grown by the slow solvent evaporation method (at acidic pH and positive electrical conductivity) over a period ranging from 7 to 10 days. The optical quality of the crystals varied according to the divalent metal inserted in the host matrix and presented prismatic morphology, as can be observed in Figure 1. The KMn/Ni sample exhibited optical transparency in visible light and was perceptible to the naked eye while the KMn/Cu showed translucency and opacity. Regarding the latter one, the emergence of structural defects, supposedly due to a non-optimized solvent evaporation rate during crystallization, surely influenced the optical quality of the crystal. In addition, the color of each crystal was correlated to the hexa-aqua complex of the second divalent metal in the K_2_MnM^II^(SO_4_)_2_(H_2_O)_6_ structure, i.e., [Ni(H_2_O)_6_]^2+^ have an emerald green color and [Cu(H_2_O)_6_]^2+^ have a light blue color.

### 2.2. Structure Solving and Lattice Parameters

Two mixed salts crystallized in the monoclinic symmetry of the *P*2_1_/*c*-space group with two K_2_Mn_1−x_M^II^_x_(SO_4_)_2_(H_2_O)_6_ formula units per unit cell (*Z* = 2) belong to the isomorphic crystallographic family of Tutton salts. The cell parameters (Table 1) have varied as a function of the molar concentration in the crystalline lattice divalent site. The use of equimolar amounts of MnSO_4_(H_2_O) and of M^II^SO_4_(H_2_O)_n_ in the synthesis does not guarantee that, during crystallization, the divalent Mn^2+^ and M^II^ ions are present in the structure with the same molar ratio. The concentration of precursor salts in the saturated growth solution, as well as the solvent evaporation rate, are, without doubt, factors that directly influence the crystallization process (nucleation and growth) and the final metal molar ratio. The chosen hydrate metal sulfate for the synthesis is also a variable that causes an impact on the site occupancy factor due to the non-equivalence of periodic properties. In short, the crystallization process and the stoichiometric ratio between metals depend on the adopted synthesis parameters.

Important contributions also arise from the physicochemical properties of the metals, as follows. Firstly, atomic radius (non-bonded)—the atomic radii of the divalent Ni (1.97 Å) and Cu (1.95 Å) metals added to the crystal are smaller than that of Mn (2.05 Å). Therefore, Ni and Cu accommodate more easily to the crystal lattice in growing. Secondly, electronegativity χ—metals with greater electronegativity (χ(Ni) = 1.91 and (χ(Cu) = 1.90) exhibit greater occupations of divalent sites due to the greater tendencies to attract electrons when compared to Mn (χ(Mn) = 1.55). Thirdly, intermolecular interactions—the Mn(II) hexa-aqua complexes are highly unstable; however, in some Tutton salts, this metal presents stability due to hydrogen bonds, as is true in the case of crystals of (NH_4_)_2_Mn(SO_4_)_2_(H_2_O)_6_ [44] and (NH_4_)_2_Mn_0.5_Zn_0.5_(SO_4_)_2_(H_2_O)_6_ [29].

On the other hand, weak interactions that destabilize [Mn(H_2_O)_6_]^2+^ complexes, such as ion–dipole and dispersion forces, are also observed in the atomic packing of potassium Tutton salts. Then, it is expected that intermolecular ion–dipole and dispersion forces do not favor the co-crystallization of [Mn(H_2_O)_6_]^2+^ complexes with K^+^ and [SO_4_]^2−^ units in this crystallographic family. Hence, the growth of K_2_Mn(SO_4_)_2_(H_2_O)_6_ individual crystals are not favored. In contrast, equimolar amounts of K_2_SO_4_ and MnSO_4_(H_2_O) in deionized water tend to form hydrated double salts (K_2_Mn(SO_4_)_2_(H_2_O)_2_) of the kröhnkite family, which crystallize with only two coordinating H_2_O molecules and exhibit triclinic ($P\bar{1}$) or monoclinic (*P*2_1_/*c*, *C*2/*c*, or *C*2/*m*) symmetry [45].

**Figure 2:**
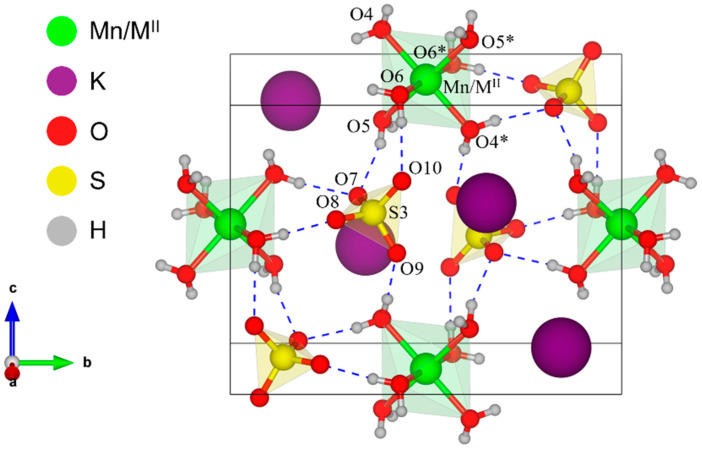
A unit cell of the mixed Tutton salts KMn/M^II^ seen on the plane *bc*. The labels followed by (*) represent the symmetric atoms of the molecular fragment.

Since the two materials were crystallographically isomorphic, the unit cell projection was the same, varying only the coordination sphere size and the metal site occupancy. Figure 2 shows a representative unit cell for mixed potassium Tutton salts visualized along the *b* and *c* planes. As can be noticed, K_2_MnM^II^(SO_4_)_2_(H_2_O)_6_ double salts are formed from three molecular fragments: slightly distorted [Mn/M^II^(H_2_O)_6_] octahedra, irregular [KO_8_] polyhedra, and [SO_4_] tetrahedra. These units interact with each other and with neighboring unit cells through O···O–H hydrogen bonds and K···O ion–dipole interactions.

The Jahn–Teller effect of axial compression is also observed in the hexacoordinated aqua complexes of the two crystals as a spontaneous geometrical distortion arising from breaking the degeneracy of *d*-orbitals. The Mn/M^II^–O bond lengths, shown in Table 2, validate the distortion, as well as the O–Mn/M^II^–O angles values shown in Table 3.

According to the data in Table 2, it can be verified that there is an axial contraction of ~0.06 Å in Mn/Ni–O4 compared to the equatorial bonds Mn/Ni–O5 and Mn/Ni–O6 for the crystal KMn/Ni; meanwhile, for the KMn/Cu crystal, this effect is more pronounced, differing on average by ~0.21 Å. Also, the equatorial coordination environment is distorted from a square with two different pairs of equatorial bonds. All axial O–Mn/M^II^–O angles are straight. Comparing the distortion occurring in the two coordination spheres, it is noted that the main differences are associated with the secondary divalent metal centers as the ligands (H_2_O molecules) and the primary metal ion (Mn^2+^) are the same for all the structures. Furthermore, the volume of the octahedron is dependent on the divalent ions.

### 2.3. Study of Intermolecular Interactions via Hirshfeld Surfaces

**Figure 3:**
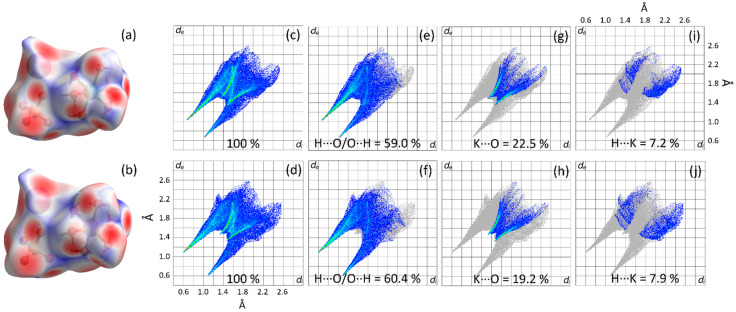
Hirshfeld surface mappings as a function of *d_norm_*: (**a**) KMn/Ni and (**b**) KMn/Cu. Full fingerprint plots: (**c**) KMn/Ni and (**d**) KMn/Cu. H···O/O···H contacts fingerprint plots: (**e**) KMn/Ni and (**f**) KMn/Cu. K···O contacts fingerprint plots: (**g**) KMn/Ni and (**h**) KMn/Cu. H···K contacts fingerprint plots: (**i**) KMn/Ni and (**j**) KMn/Cu.

Hirshfeld surface analysis was carried out to understand better the intermolecular interactions in the KMn/M^II^ crystals. Figure 3a,b exhibit the occupation of the forming units. The crystal lattice is divided into three ionic fragments, K^+^, [Mn/M^II^(H_2_O)_6_]^2+^, and [SO_4_]^2−^, in which they establish contacts stabilizing the packing of crystalline structures.

The Hirshfeld surfaces are illustrated using a 3D electron density map as a function of *d_norm_*. The color scheme corresponds to the intensity of each intermolecular contact: regions in red represent interactions with distances shorter than the van der Waals radii, regions in white characterize contacts equal to the van der Waals radii, and regions in blue are associated with intermolecular interactions with distances greater than van der Waals radii [46,47]. A very similar pattern of Hirshfeld surfaces is established because the two salts belong to the isomorphic Tutton crystal class. In both compounds, the red areas around oxygen, hydrogen, and potassium atoms are predominant and indicate stronger interactions related to H···O/O···H, K···O and H···K close contacts.

The Hirshfeld analysis can also be displayed as 2D fingerprints of the different types of contacts that occur in a crystalline system. The 2D fingerprints are shown in Figure 3c–j as histograms in terms of the *d_e_* and *d_i_* functions that denote a fraction of colored points on the surface, where the specific close contacts and the distant ones are represented by the red and blue areas, respectively [48].

Figure 3c,d show 2D fingerprint plots of the 100% cumulative for the KMn/Ni and KMn/Cu Tuttons, respectively. Significant similarities are observed due to the isomorphism of these materials. However, some differences are noticed at low values of *d_e_* and *d_i_* because of the variation of divalent metals in the M^II^ sites of the structures. The cumulative patterns are divided into three stratified histograms (Figure 3e–j) that correspond to H···O/O···H, K···O, and H···K interactions with their corresponding percentage contributions. The H···O/O···H intermolecular interactions contribute to more than 59.0% of the Hirshfeld surface. The most intense and closest contact occurs between the ions, where the positively charged region of the surface corresponds to the hydrogen atoms of the H_2_O molecules. The negative pole corresponds to the oxygen atoms of the [SO_4_] units. Therefore, this is the main contact stabilizing the KMn/M^II^ Tutton salts crystal lattice. Furthermore, the sharp and reddish peaks seen in the histograms of Figure 3e,f at low values of *d_e_* and *d_i_* corroborate the fact; thus, these interactions arise from strong intermolecular forces.

On the other hand, the K···O and H···K ion–dipole intermolecular interactions, represented in Figure 3g–j, are accounted as intermediate contacts. The insertion of different metals into the framework causes slight changes in the intermolecular interactions, favoring some specific contacts. For example, the KMn/Ni crystal pattern presents 22.5% of K···O and 7.2% of H···K interactions while, for the KMn/Cu crystal, they reach 19.2% and 7.9%, respectively. The K^+^, [SO_4_]^2−^, and [M(H_2_O)_6_]^2+^ ionic fragments are then co-crystallized in the K_2_MnM^II^(SO_4_)_2_(H_2_O)_6_ structure and stabilized via hydrogen bonds with contributions from ion–dipole interactions.

Weaker intermolecular contacts by dispersion forces are also observed in the KMn/Ni and KMn/Cu structures, such as H···H and O···O [43]. In addition, the calculated differences between the compounds may be correlated with the XRD results, where differences in the lattice parameters are also documented and attributed to the random occupation factor of equivalent crystallographic sites (divalent sites) by the distinct metal cations.

### 2.4. Crystal Voids

**Figure 4:**
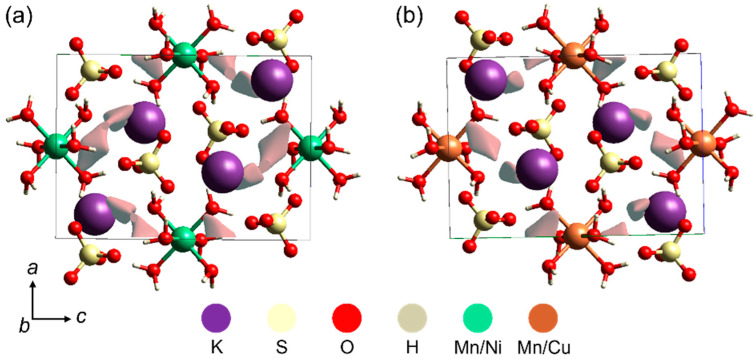
Calculated voids (seen along the unit cell *b*-axis) for the mixed potassium Tutton crystals: (**a**) KMn/Ni and (**b**) KMn/Cu.

Further computational analysis of the crystal structures was carried out to calculate the voids based on the unit cell parameters. For this, the calculation of electronic density isosurfaces was considered through the promolecule density approach, which makes it possible to compute the volume of voids, their surface area, and the percentage of free space in crystalline structures, as represented in Figure 4a,b.

The KMn/Ni and KMn/Cu crystals have lower percentages of free spaces in the unit cells: 1.64% (10.70 Å^3^) and 1.11% (7.32 Å^3^), respectively. Isosurface areas were also calculated and are listed in Table 4. Since the areas are proportional to the percentage of voids, the KMn/Cu crystal has a smaller surface area (46.47 Å^2^) than the KMn/Ni crystal (60.70 Å^2^). These results must be taken into account in the case of the introduction of doping impurities to modulate specific properties of crystals. It is necessary to consider the volume and the ionic radius of species that may be added as an interstitial impurity.

### 2.5. Calculated Properties of the Hexa-Aqua Transition Metal Complexes

The octahedral hexa-aqua coordination complexes, Mn(II), Ni(II), and Cu(II), present in mixed Tutton salts, were subjected to DFT calculations to help elucidate the Jahn–Teller effect observed in the crystal structures. The geometries of [Mn(H_2_O)_6_]^2+^ and [Ni(H_2_O)_6_] ^2+^ were optimized at several spin multiplicities to determine and confirm the ground state of each complex and study the occurrence of the effect.

For the [Ni(H_2_O)_6_]^2+^ complex, the results reveal that the optimized structure with triplet spin multiplicity has lower energy than the optimized structure with singlet spin multiplicity by 23.91 kcal/mol of *E_total_* and 30.73 kcal/mol and 28.06 kcal/mol in terms of *G*^298^ and *E*_ZPVE_, respectively. On the other hand, the geometry optimizations of the [Mn(H_2_O)_6_]^2+^ complex indicate that the ground state with sextet spin multiplicity is more energetically favorable than the state with quartet spin multiplicity by 47.99 kcal/mol of *E_total_* and 48.78 kcal/mol and 48.43 kcal/mol in terms of *G*^298^ and *E*_ZPVE_, respectively.

The thermodynamic parameters associated with the complexation in the formation of the studied complexes are also investigated. The Gibbs free energy (Δ_coord_*G*^298^), enthalpy (Δ_coord_*H*), and electronic energy corrected by zero-point vibrational energy (Δ_coord_*E*_ZPVE_) are calculated in the gas phase. These key thermodynamic properties are obtained in terms of the parameters calculated separately for the moieties of each complex in accordance with Equation (1):

(1) $$\Delta_{\mathrm{coord}}E=E_{\mathrm{coord}}{\left[M{\left(H_{2}O\right)}_{6}\right]}^{2+}-\left[6E(H_{2}O)+E{\left(M\right)}^{2+}\right]$$

where *E* refers to *G*^298^, *H*, or *E*_ZPVE_.

The calculated thermodynamic properties are listed in Table 5. From these values, one can observe that the complexation process is exothermic and spontaneous in all three complexes studied, considering different possible spin multiplicity values.

Thus, the states with doublet, triplet, and sextet spin multiplicities are the ground states of the complexes [Cu(H_2_O)_6_]^2+^, [Ni(H_2_O)_6_]^2+^ and [Mn(H_2_O)_6_]^2+^, respectively. In order to reach the lowest energy, the *e_g_* orbital degeneracy is broken, leading to a tetragonal distortion (*D*_4h_) from octahedral geometry (*O*_h_), which is indicative of the Jahn–Teller effect. Such distortions are observed for the [Cu(H_2_O)_6_]^2+^ complex and for the [Mn(H_2_O)_6_]^2+^ complex with quartet spin multiplicity, both exhibiting axial elongation of the M–O bond length. The ground state [Ni(H_2_O)_6_]^2+^ and [Mn(H_2_O)_6_]^2+^ complexes with triplet and sextet spin multiplicity, respectively, present a regular octahedral geometry.

The structural parameters related to the bond lengths and bond angles of these hexa-aqua metal complexes were also analyzed and based on the optimized geometries (see Table 6). The coordination environment is highly sensitive to the spin multiplicity of the metal in the complex, where the main distinction can be described in terms of axial M–O bond length variations, which are associated with the Jahn–Teller effects.

The [Cu(H_2_O)_6_]^2+^ complex presents the most pronounced calculated axial elongation, with an axial Cu–O bond length of 2.28 Å, which is longer by about ~0.27 Å relative to the equatorial M–O bond lengths. The structural parameters obtained from the optimized geometry results are in good agreement with those obtained from XRD experiments where, in the KMn/Cu crystals, the axial bonds were longer on average by ~0.21 Å. The [Mn(H_2_O)_6_]^2+^ complex with a quartet spin multiplicity shows a pronounced axial distortion compared with equatorial bond lengths, differing on average by ~0.20 Å, comparable to that observed in the crystal structure. Also, the equatorial bonds feature a parallelogram-like distortion, which is also observed in the crystal structure (Table 2). The ground state of the [Mn(H_2_O)_6_]^2+^ complex with a sextet spin multiplicity is undistorted. The singlet spin multiplicity for the [Ni(H_2_O)_6_]^2+^ complex converges to a square planar structure without axial water ligands while a triplet spin multiplicity leads to an octahedral structure, as observed experimentally by XRD.

**Figure 5:**
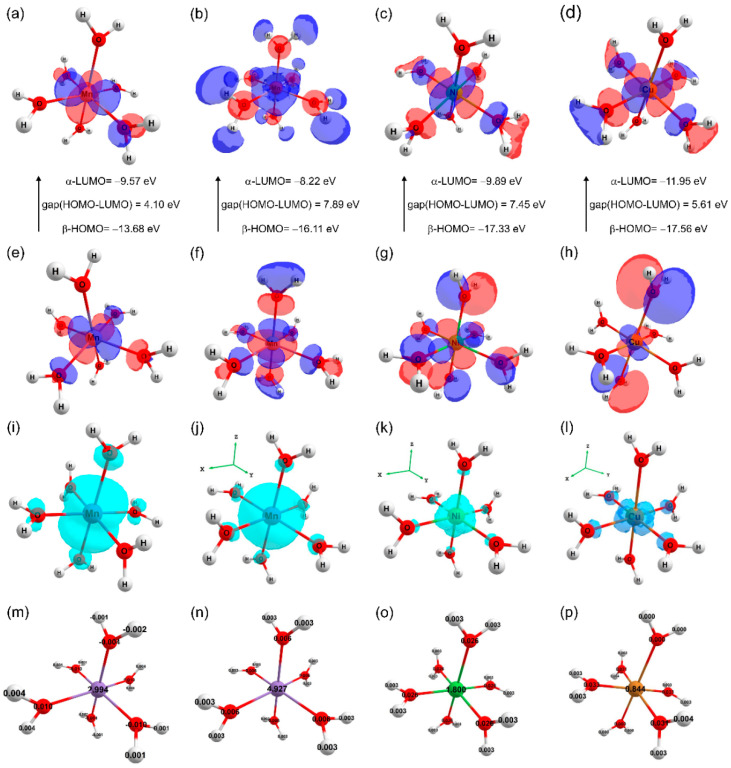
Left-to-right: quartet and sextet [Mn(H_2_O)_6_]^2+^, triplet [Ni(H_2_O)_6_]^2+^, and doublet [Cu(H_2_O)_6_]^2+^. (**a**–**d**) LUMO and (**e**–**h**) HOMO with HOMO–LUMO energy gaps; (**i**–**l**) spin density maps, and (**m**–**p**) atomic spin values.

Since M–O bonds have a highly ionic character and H_2_O is a weak field ligand [39], the mixing between 3*d^n^* orbitals and 2*p* orbitals is low, contributing to a high-spin occupancy, where the *t*_2*g*_ and *e_g_* are non-bonding metal-centered orbitals [35]. In this aspect, the Jahn–Teller effect in the octahedral structure distortion of metal complexes occurring in [Cu(H_2_O)_6_]^2+^ and the [Mn(H_2_O)_6_]^2+^ with quartet spin multiplicity, can be interpreted in terms of the electronic configuration of an odd number of electrons in *e_g_* orbitals [38]. The characteristics of the spin densities, partial atomic charge, and frontier molecular orbital of the metal complexes are explored to clarify such a distortion. The results are listed in Table 7 and presented in Figure 5.

The spin density represents the unpaired electron density distribution. For the [Ni(H_2_O)_6_]^2+^ and [Mn(H_2_O)_6_]^2+^ complexes with triplet and sextet spin multiplicities, respectively, a symmetric spin density distribution is observed (Table 7, Figure 5j,k) with 1.80 and 4.93 of electron density, respectively, centered in the divalent metal atoms. In contrast, for the complexes [Cu(H_2_O)_6_]^2+^ with doublet spin multiplicity and [Mn(H_2_O)_6_]^2+^ with quartet spin multiplicity, asymmetric spin density distributions are observed (Figure 5i) with 0.84 and 2.99 of electron density, respectively, localized on the divalent metal atoms. These asymmetric spin densities at the metal are also reflected in asymmetric residual spin densities on the ligand O atoms.

The electron intake of metal ions from ligand molecules in a complex formation suggests a charge-transfer process, as quantified by the difference between the free metal charge and the metal charge in a hexa-aqua complex. A symmetric Mulliken partial atomic charge distribution over H_2_O ligands is observed in the triplet and sextet states of the [Ni(H_2_O)_6_]^2+^ and [Mn(H_2_O)_6_]^2+^ complexes, respectively. The charge in the metal center of the triplet hexa-aqua nickel(II) complex is equal to 1.04|*e*|, indicating an electron gain of 0.96|*e*|. Also, the charge in the metal center of the sextet hexa-aqua manganese(II) complex is equal to 1.11|*e*|, revealing an intake of 0.89|*e*|. An asymmetric distribution of Mulliken partial atomic charges over the coordinated H_2_O ligands is noticed in the doublet and quartet states of the [Cu(H_2_O)_6_]^2+^ and [Mn(H_2_O)_6_]^2+^ complexes, respectively, highlighting an uneven metal–ligand electronic transfer. Additionally, the charge on the metal center of the doublet hexa-aqua copper(II) complex is equal to 0.98|*e*| and indicates an electron gain of 1.02|*e*|; meanwhile, in the quartet state of the hexa-aqua manganese(II) complex, the metal center charge is equal to 1.02|*e*|, with an intake of 0.98|*e*|.

The highest occupied molecular orbital (HOMO) and lowest unoccupied molecular orbital (LUMO) were calculated for each hexa-aqua metal complex investigated here, with their energy values listed in Table 7 and spatial distributions illustrated in Figure 5. Since the hexa-aqua metal complexes have unpaired electrons, they are treated as open-shell systems calculated with a double determinant and their molecular orbitals are defined in terms of *alfa* (α) and *beta* (β) orbitals. The results show that the [Cu(H_2_O)_6_]^2+^ complexes with a doublet spin multiplicity present HOMO and LUMO β orbitals values (Figure 5d,h) of −17.56 eV and −11.95 eV, respectively, which define the HOMO–LUMO energy gap (GHL) of 5.61 eV.

The sextet and triplet states of the [Mn(H_2_O)_6_]^2+^ and Ni(H_2_O)_6_]^2+^ complexes exhibit higher GHLs, with 7.89 eV and 7.45 eV, respectively (Figure 5b,f and Figure 5c,g), while the quartet spin state of the [Mn(H_2_O)_6_]^2+^ has the lowest GHL of 4.10 eV. The lowest energy GHL of the quartet manganese hexa-aqua complex, followed by the doublet copper hexa-aqua complex, reveals a low electronic kinetic stability and a high chemical reactivity, highlighting that metal–ligand bonds are dominated by charge-transfer interactions.

### 2.6. Thermal Properties

**Figure 6:**
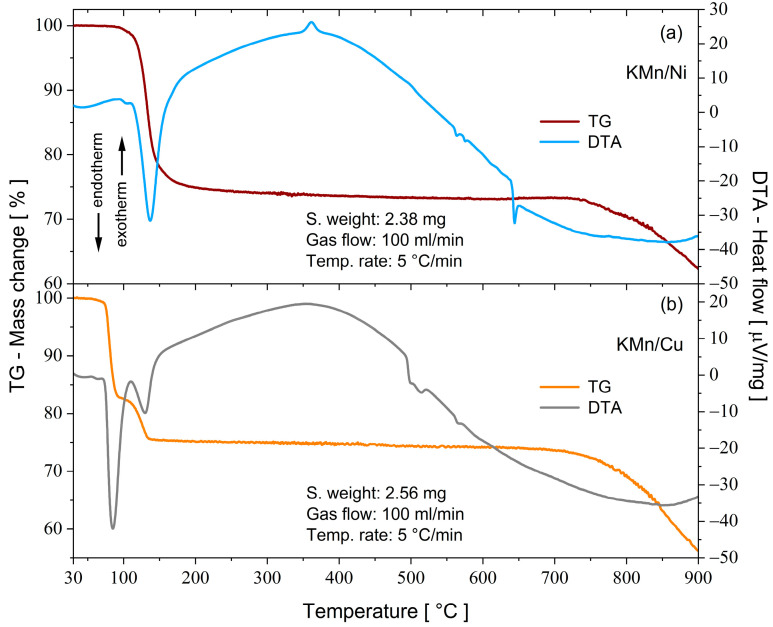
Simultaneous TG-DTA thermograms of the mixed Tutton salts: (**a**) KMn/Ni and (**b**) KMn/Cu.

Figure 6a,b depict the TG-DTA thermograms in the 30–900 °C temperature range for KMn/M^II^ Tutton salts. The TG curves show that the KMn/Ni and KMn/Cu samples are thermally stable at around 96 and 68 °C, respectively. The difference in stability between the samples is associated with the substitution effect that the different divalent cations cause in the coordination and intermolecular interactions in unit cells. Above these temperatures, each salt exhibits mass loss due to the release of H_2_O molecules. For the KMn/Cu crystal, the dehydration stage is distributed into two intervals: the first characterizes the evaporation of 4.2, and the second 1.8, H_2_O molecules. In contrast, the dehydration process for the KMn/Ni is verified only in a single stage with the release of 6.0 coordinating H_2_O molecules. The endothermal events in the DTA curves are consistent with the dehydration of the salts and indicate phase transformations from the hydrated structures to the anhydrous phases. Table 8 summarizes the types of thermal events observed for both samples, as well as the values of the temperatures, mass losses, and molecular fragments.

After both samples undergo structural transformation to the anhydrous form, the new phases remain stable up to 733 °C for the KMn/Ni crystal and 720 °C for the KMn/Cu crystal. As illustrated in Table 8, some physical events were analyzed from the DTA curves. At around 361 °C, the KMn/Ni sample thermogram shows a broad endothermal peak characteristic of a phase transition from an anhydrous amorphous structure to an anhydrous crystalline one, similar to the one reported by A. Morales et al. [49]. In the KMn/Ni sample, the dehydration process causes the breakdown of numerous intermolecular interactions and makes the amorphous system. With increasing temperature, primary chemical bonds are favored and promote a crystallization of the amorphous system.

At higher temperatures (>490 °C), other endothermal events are observed for the two mixed crystals. For the KMn/Ni, at around 563 and 575 °C, two events of low heat flux are verified and possibly associated with a solid–solid transition from K_2_SO_4_ in the anhydrous form of the system K_2_Mn_0.03_Ni_0.97_(SO_4_)_2_. There is a structural change from β-K_2_SO_4_ (orthorhombic) to α-K_2_SO_4_ (trigonal), as determined in other studies involving the K_2_SO_4_ compound as a constituent [13]. These same events are also detected in the DTA curve for the KMn/Cu sample and are attributed to the same process. On the other hand, the difference between the transition temperatures may be associated with the structural stability that the coordination complexes promote in the crystal lattice, consistent with the DFT calculations. The events at 565 and 644 °C for the KMn/Cu and KMn/Ni samples, respectively, correspond to a first-order phenomenon attributed to the initial melting of MnSO_4_ [50]. Final vitreous residues with percentages of 62.4% for the KMn/Ni and 56.2% for the KMn/Cu were determined at 900 °C.

**Figure 7:**
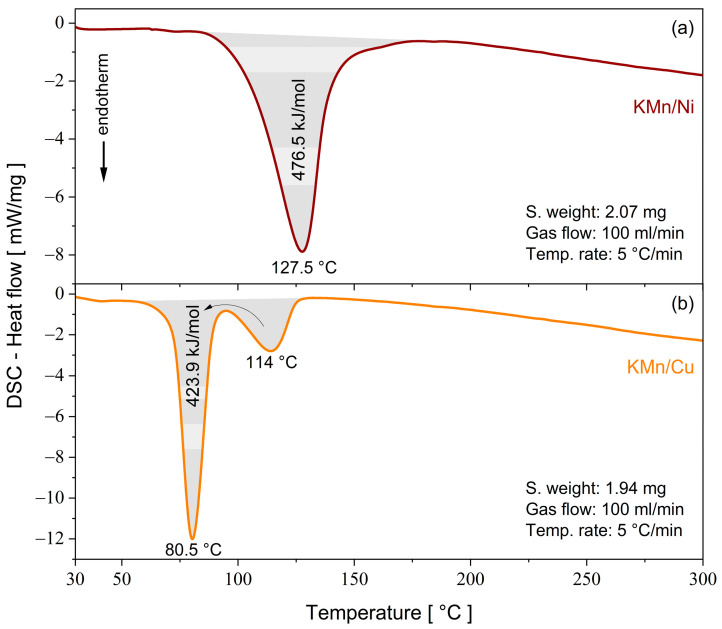
DSC curves of the mixed Tutton salts: (**a**) KMn/Ni and (**b**) KMn/Cu.

A complementary thermal experiment by means of DSC analysis (Figure 7) was conducted to evaluate the amount of heat involved in the dehydration process from the enthalpy reaction (Δ*H*). The values estimated from the deconvolution of the endothermal peaks presented energies of 476.5 and 423.9 kJ/mol for the KMn/Ni and KMn/Cu crystals, respectively. Due to its greater structural and thermal stability, the KMn/Ni salt requires greater energy to break the hydrogen bonds, as previously predicted by the theoretical data of Δ*G*^298^, Δ*H*, and Δ*E*_ZPVE_ (see Table 5).

To verify the possibility of the novel mixed Tutton salts being applied as materials for thermochemical heat storage devices, the parameter of storage energy density per volume (in J/m^3^) was evaluated using the enthalpy of dehydration through the following equation:

(2) $$\Delta H_{V}=\frac{\Delta H_{\exp}}{{\mathrm{MM}}_{S}}\rho_{S}$$

where Δ*H*_exp_ is the experimental enthalpy, MM_TS_ is the total molecular mass of the salt, and *ρ*_S_ is the volumetric mass (KMn/Ni: 2.232 g/cm^3^ and KMn/Cu: 2.191 g/cm^3^).

As seen in Table 9, the novel mixed Tutton salts KMn/M^II^ meet the criteria of dehydration temperature (<120 °C) and energy density (≥1.3 GJ/m^3^) [2] as promising thermochemical materials for residential applications. When compared to similar individual compounds reported in the literature [3], such as K_2_Ni(SO_4_)_2_(H_2_O)_6_ (1.78 GJ/m^3^) and K_2_Cu(SO_4_)_2_(H_2_O)_6_ (1.97 GJ/m^3^), the results here are significantly higher. Although t is not completely clear why the energy density values of the new salts are higher than those previously reported, these can be attributed to the different coordination sphere sizes, different site occupations, structural and thermal stability, and structural defect density. Different types of bonds (weak, strong, and dangling bonds) involved in the crystalline lattice impart different thermal and structural properties.

## 3. Experimental and Theoretical Procedures

### 3.1. Single-Crystal Synthesis

Mixed Tutton salts with the chemical composition K_2_Mn_1−x_M^II^_x_(SO_4_)_2_(H_2_O)_6_, where M^II^ = Ni and Cu, were crystallized from saturated solutions by the slow evaporation method. For the synthesis, the precursor reagents (Sigma Aldrich > 99%, Darmstadt, Germany), with their respective amounts listed in Table 10, were homogenized in a 50 mL aqueous solution by magnetic stirring at 360 RPM under a constant temperature of 50 °C for 400 min. The final solutions with an acidic pH≈ of 3.6 were filtered to remove solid impurities, covered with a plastic film containing twelve orifices, randomly distributed, and stored in an oven at a constant temperature of 35 °C.

### 3.2. Structural and Thermal Characterization

Single crystals were analyzed on a Bruker D8 Venture microsource diffractometer with a Photon II CPAD detector and an ImS 3.0 Incoatec MoK_α_ (λ = 0.71073 Å) microfocus source. The samples were kept at a constant temperature of 301 K during data collection using an Oxford Cryostream cryostat (800 series Cryostream Plus) attached to the system. The APEX 4 software was used for data collection [51]. The data fitting and the unit cell refinement were conducted using the Bruker SAINT+ package [52]. Numerical absorption correction was performed with the SADABS software (version 2.03) [53]. From the Olex2 interface program to the SHELX suite [54], the structure was solved by the intrinsic phasing method implemented in ShelXT, allowing the identification of the position of most of the non-hydrogen atoms [55]. The remaining non-hydrogen atoms were localized from different Fourier maps calculated from full-matrix least-squares refinement cycles on *F*^2^ with ShelLX and refined using anisotropic displacement parameters [56]. Hydrogen atoms were inserted at calculated positions and analyzed using a riding model. The MERCURY program was used to visualize the crystallographic structure and generate the CIF information file [57]. The CIF files were deposited in the Cambridge Structural Database (CSD) under the codes 220517 (KMn/Ni) and 220516 (KMn/Cu).

The thermoanalytical techniques of thermogravimetry (TG), differential thermal analysis (DTA), and differential scanning calorimetry (DSC) were applied to this study using DTG-60 and DSC-60 thermal analyzers, both from Shimadzu. For the TG-DTA experiments, samples in powder form were distributed on the bottom of platinum crucibles and heated in the temperature range between 30 and 900 °C, with a flow rate of 5 °C/min and a nitrogen atmosphere (100 mL/min). For the DSC experiments, the powder samples were uniformly distributed on an alumina crucible. The analyses in the temperature range from 30 to 500 °C were performed using a 5 °C/min heating rate in a nitrogen atmosphere with a flow rate of 100 mL/min.

### 3.3. Computational Procedure

The DFT calculations of the hexacoordinated metal complexes with H_2_O molecules as ligands were performed using the PBE1PBE functional [40]. The application of this functional is justified by its widespread use in the study of coordination complexes of first-row transition metals, providing structural and electronic properties in good agreement with experimental results [39,58]. The metals, H, and O atoms were treated with the quadruple-ζ QZVP basis set [59]. The two electron integrals and their derivatives were conducted using a pruned grid containing 75 radial shells and 302 angular points per shell. The ground state of each complex, considering the divalent character of each metal in [M(H_2_O)_6_]^2+^ in accordance with their *d^n^* electronic configuration, was treated regarding their particular spin multiplicity given by *s* = 2*n* + 1, where *n* is the number of unpaired electrons times the electronic spin value of 1/2. Thus, the fully optimized geometry of each complex was obtained for the following *d*-orbital population and spin multiplicity: [Mn(H_2_O)_6_]^2+^ (3*d*^5^; *s* = 4 and 6), [Ni(H_2_O)_6_]^2+^ (3*d*^8^; *s* = 1 and 3), and [Cu(H_2_O)_6_]^2+^ (3*d*^9^; *s* = 2). The optimized geometries were confirmed as a minimum in the potential energy surface by ascertaining that all calculated vibrational frequencies were positive.

The DFT calculations were performed using the software Gaussian16 [60], considering all the complexes as unrestricted open-shell systems. The results of the electronic structure calculations were analyzed using the software ChemCraft 1.8 [61] and discussed in the context of findings from the experiments.

### 3.4. Hirshfeld Surface Analysis and Unit Cell Voids

The Hirshfeld 3D surfaces were plotted using the Crystal Explorer 17.5 software [62], as a function of the normalized distance (*d*_norm_) that is based on contact distances between the nearest atoms inside (*d*_i_) and external (*d*_e_) to the surface and expressed as *d*_norm_= (*d*_i_ − *r*_i_^vdw^/*r*_i_^vdw^)/(*d*_e_ − *r*_e_^vdw^/*r*_e_^vdw^); in which, *r*_i_^vdw^ and *r*_e_^vdw^ are the van der Waals radii of the appropriate atoms internal and external to the surface, respectively. The surfaces were generated considering the promolecule electron density approach, coming from the tabulation of the atomic wavefunction expanded using the Thakkar basis set for ab initio calculation, taking 0.002 a.u. as a typical cutoff density standard setting [63]. The color pattern established in blue, white, and red corresponds to more distant, intermediate, and closer contacts to the van der Walls radius, respectively [64].

Additionally, the 2D fingerprint plots were extracted from these models, which are presented as a function of the distance from a given point on the Hirshfeld surface to *d*_e_ and the distance from a given point on the Hirshfeld surface to *d*_i_ [65]. Crystal voids were also estimated using the Crystal Explorer 17 software, based on electronic density isosurfaces (0.002 a. u.) generated inside the primitive unit cell of each Tutton salt [66].

## 4. Conclusions

In this paper, two novel mixed Tutton salts with the chemical formulas K_2_Mn_0.03_Ni_0.97_(SO_4_)_2_(H_2_O)_6_—KMn/Ni and K_2_Mn_0.18_Cu_0.82_(SO_4_)_2_(H_2_O)_6_—KMn/Cu were successfully grown by the slow solvent evaporation method and investigated as compounds for thermochemical heat storage systems. Both salts crystallize in monoclinic symmetry (*P*2_1_/*c*) with two bivalent cations occupying the same type of site in the unit cell. The crystallographic structures were deposited in the Cambridge Structural Database under the numbers 220517 and 220516 for the K_2_Mn_0.03_Ni_0.97_(SO_4_)_2_(H_2_O)_6_ and K_2_Mn_0.18_Cu_0.82_(SO_4_)_2_(H_2_O)_6_ samples, respectively. Hirshfeld surfaces and 2D fingerprints were calculated to elucidate the intermolecular interactions involved between the molecular fragments of the crystals. It was found that both systems are stabilized by hydrogen bonds (H···O/O···H) and dipole–ion interactions (K···O and H···O). From electronic isosurfaces, crystal voids were evaluated to estimate the unit cell free volume, surface area, and void percentage of the solids. The theoretical studies performed by DFT calculations helped to elucidate the electronic, structural, and thermodynamic properties of the metal complex aqua-hexa coordinated presented in the mixed crystals. The thermodynamics findings, presented in terms of Δ_coord_*G*^298^, Δ_coord_*H*, and Δ_coord_*E*_ZPVE_, revealed that all metal complexes investigated are chemically stable. The structural parameters of each complex were optimized and the results showed good agreement with those determined by XRD. Bond lengths and angles (calculated and experimental), as well as their deviations, were correlated in terms of electronic effects associated with spin densities and partial atomic charges, where octahedral distortions were treated in the context of the Jahn–Teller effect. The HOMO and LUMO orbitals of triplet and sextet spin multiplicities for the [Ni(H_2_O)_6_]^2+^ and [Mn(H_2_O)_6_]^2+^ complexes, respectively, and their energy gaps, indicate that these electronic configurations yield kinetically stable complexes. The [Mn(H_2_O)_6_]^2+^ complex with quadruplet spin multiplicity had the lowest energy gap, followed by the [Mn(H_2_O)_6_]^2+^ complex with doublet spin multiplicity, which revealed their low kinetic stability and enhanced chemical reactivity. Thermoanalytical measurements showed that the KMn/Ni and KMn/Cu crystals have thermal stability up to 96 and 68 °C, respectively. Above these temperatures, the crystals undergo successive structural changes related to the processes of dehydration, crystallization, solid–solid transition, and melting. From the dehydration enthalpy values, estimated from DSC curves, it was verified that both samples had the potential for use in thermochemical heat storage devices due to their high energy density, calculated as 2.43 GJ/m^3^ for KMn/Ni and 2.14 GJ/m^3^ for KMn/Cu. Therefore, these new mixed potassium Tutton salts show promising structural, electronic, and thermal properties for developing energy-storing materials.

## Figures and Tables

**Table 1:** XRD crystallographic determination of the KMn/Ni and KMn/Cu Tutton salts.

Empirical Formula	H_12_K_2_Mn_0.03_Ni_0.97_O_14_S_2_	H_12_K_2_Mn_0.18_Cu_0.82_O_14_S_2_
Formula weight [g/mol]	437.01	434.32
Temperature [K]	301.00	302.00
Crystal system	monoclinic	monoclinic
Space group	*P*2_1_/c	*P*2_1_/c
*a* [Å]	6.1351(2)	6.1690(2)
*b* [Å]	12.1900(3)	12.1406(5)
*c* [Å]	9.0039(3)	9.0784(3)
α [°]	90	90
β [°]	105.0400(10)	104.4720(10)
γ [°]	90	90
Volume [Å^3^]	650.31(3)	658.36(4)
Z	2	2
ρ_Calc_ [g/cm^3^]	2.232	2.191
μ [mm^−1^]	2.507	2.553
*F*(000)	444.0	433.0
Crystal size [mm^3^]	0.518 × 0.227 × 0.153	1.105 × 0.511 × 0.267
Radiation	MoK_α_ (λ = 0.71073)	MoK_α_ (λ = 0.71073)
2θ range for data collection [°]	5.754 to 84.544	5.722 to 59.982
Index ranges	−11 ≤ *h* ≤ 9, −22 ≤ *k* ≤ 20, −14 ≤ *l* ≤ 16	−8 ≤ *h* ≤ 8, −17 ≤ *k* ≤ 17, −12 ≤ *l* ≤ 12
Reflections collected	26,978	17,974
Independent reflections	4046 [R_int_ = 0.0347, R_sigma_ = 0.0246]	1912 [R_int_ = 0.0406, R_sigma_ = 0.0261]
Data/restraints/parameters	4046/0/93	1912/0/90
Goodness-of-fit on *F*^2^	1.045	1.081
Final R indexes [I ≥ 2σ (I)]	R_1_ = 0.0254, wR_2_ = 0.0620	R_1_ = 0.0244, wR_2_ = 0.0650
Final R indexes [all data ]	R_1_ = 0.0313, wR_2_ = 0.0649	R_1_ = 0.0247, wR_2_ = 0.0653
Largest diff. peak/hole [Å^−3^]	0.52/−0.48	0.58/−0.41

**Table 2:** Bond lengths determined using X-ray crystallography.

KMn/Ni		KMn/Cu	
Atoms	Length [Å]	Atoms	Length [Å]
Mn/Ni–O4	2.021(7)	Mn/Cu–O4	1.961(11)
Mn/Ni–O5	2.086(7)	Mn/Cu–O5	2.095(12)
Mn/Ni–O6	2.080(7)	Mn/Cu–O6	2.248(12)
S3–O7	1.475(7)	S3–O7	1.478(12)
S3–O8	1.481(7)	S3–O8	1.481(11)
S3–O9	1.478(8)	S3–O9	1.474(12)
S3–O10	1.465(9)	S3–O10	1.464(13)

**Table 3:** Bond angles determined using X-ray crystallography.

KMn/Ni		KMn/Cu	
Atoms	Angle [°]	Atoms	Angle [°]
O4–Mn/Ni–O4*	180.0(5)	O4–Mn/Cu–O4†	180.0(5)
O4–Mn/Ni–O5*	90.0(3)	O4–Mn/Cu–O5†	89.6(5)
O4*–Mn/Ni–O5*	89.9(3)	O4†–Mn/Cu–O5†	90.3(5)
O4–Mn/Ni–O5	90.0(3)	O4–Mn/Cu–O5	89.6(5)
O4*–Mn/Ni–O5	89.9(3)	O4†–Mn/Cu–O5	90.3(5)
O4*–Mn/Ni–O6*	90.3(3)	O4†–Mn/Cu–O6†	89.3(4)
O4–Mn/Ni–O6	89.6(3)	O4–Mn/Cu–O6	90.6(4)
O4–Mn/Ni–O6*	89.6(3)	O4–Mn/Cu–O6†	90.6(4)
O4*–Mn/Ni–O6	90.3(3)	O4†–Mn/Cu–O6	89.3(4)
O5–Mn/Ni–O5*	180.0(4)	O5–Mn/Cu–O5†	180.0(4)
O5*–Mn/Ni–O6*	91.6(3)	O5†–Mn/Cu–O6†	90.6(5)
O5–Mn/Ni–O6*	91.6(3)	O5–Mn/Cu–O6†	89.4(5)
O5*–Mn/Ni–O6	88.3(3)	O5†–Mn/Cu–O6	89.4(5)
O5–Mn/Ni–O6	88.3(3)	O5–Mn/Cu–O6	90.5(5)
O6–Mn/Ni–O6*	180.0(5)	O6–Mn/Cu–O6†	180.0(5)
O7–S3–O8	110.2(4)	O7–S3–O8	110.0(7)
O7–S3–O9	108.0(5)	O7–S3–O9	110.0(8)
O9–S3–O8	109.9(5)	O9–S3–O8	108.0(7)
O7–S3–O10	108.4(5)	O7–S3–O10	110.4(8)
O10–S3–O8	110.6(5)	O10–S3–O8	108.2(8)
O10–S3–O9	109.5(6)	O10–S3–O9	109.8(9)

* 1 − x, −y, 1 − z; † 1 − x, 2 − y, 1 − z.

**Table 4:** Structural parameters associated with the voids analysis in the unit cell of mixed Tutton salts.

Crystal	Unit Cell Volume [Å^3^]	Voids Volume [Å^3^]	Voids Percentage [%]	Voids Surface Area [Å^2^]
KMn/Ni	650.31	10.70	1.64	60.70
KMn/Cu	658.36	7.32	1.11	46.47

**Table 5:** Calculated thermodynamic properties of metal complexes.

Complex	Spin Multiplicity	Δ_coord_*G*^298^ [kcal/mol]	Δ_coord_*H* [kcal/mol]	Δ_coord_*E*_ZPVE_ [kcal/mol]
[Cu(H_2_O)_6_]^2+^	2	−296.00	−351.49	−347.75
[Ni(H_2_O)_6_]^2+^	3	−296.00	−352.03	−348.18
[Ni(H_2_O)_4_]^2+^	1	−339.45	−400.05	−393.65
[Mn(H_2_O)_6_]^2+^	6	−242.20	−296.65	−293.17
[Mn(H_2_O)_6_]^2+^	4	−274.90	−329.57	−325.99

**Table 6:** Geometric parameters calculated for the metal complexes.

Complex	Spin Multiplicity	M–O_ax_ [Å]	O–M–O_ax_ [°]	M–O_eq1_ [Å]	M–O_eq2_ [Å]	O–M–O_eq_ [°]
[Cu(H_2_O)_6_]^2+^	2	2.28	89.97	2.01	2.00	90.03
[Ni(H_2_O)_6_]^2+^	3	2.07	89.99	2.07	2.07	90.01
[Ni(H_2_O)_4_]^2+^	1	--	--	1.88	1.88	91.54
[Mn(H_2_O)_6_]^2+^	6	2.20	89.99	2.20	2.20	90.01
[Mn(H_2_O)_6_]^2+^	4	2.26	89.93	2.02	2.10	91.38

**Table 7:** Electronic properties calculated regarding metal complexes.

Complex	*q*(O)_ax_	*q*(O)_eq1_	*q*(O)_eq2_	*q*(M)	*ρ_s__*_ax_(O)	*ρ_s__*_eq1_(O)	*ρ_s__*_eq2_(O)	*ρ_s_*(M)	HOMO [eV]	LUMO [eV]	Gap [eV]
[Cu(H_2_O)_6_]^2+^ *s* = 2	−0.39	−0.37	−0.34	0.98	0.00	0.03	0.03	0.84	−17.56	−11.95	5.61
[Ni(H_2_O)_6_]^2+^ *s* = 3	−0.38	−0.38	−0.38	1.04	0.03	0.03	0.03	1.80	−17.33	−9.89	7.45
[Ni(H_2_O)_4_]^2+^ *s* = 1	--	−0.35	−0.35	1.02	--	--	--	--	−16.78	−11.31	5.48
[Mn(H_2_O)_6_]^2+^ *s* = 6	−0.39	−0.39	−0.39	1.11	0.01	0.01	0.01	4.93	−16.11	−8.22	7.89
[Mn(H_2_O)_6_]^2+^ *s* = 4	−0.39	−0.36	−0.38	1.02	0.01	0.00	−0.01	2.99	−13.68	−9.57	4.10

**Table 8:** Fragmentation events observed for KMn/M^II^ Tutton salts in TG-DTA thermograms.

Sample	Temperature [°C]	TG				DTA [°C]	
		Weight Loss [%]	Weight Loss [mg]	Molar Mass [g·mol^−1^]	Molecular Fragment	[↓] Endothermal [↑] Exothermal	Event Type
KMn/Ni	30–200	25.0	0.59	109.2	6.0(H_2_O)	101.8 ↓ 137.2 ↓	dehydration dehydration
	200–900	12.6	0.30	55.2	SO + 0.3O	361.7 ↑ 563.4 ↓ 575.6 ↓ 644.8 ↓	crystallization phase transition -- MnSO_4_ melting
KMn/Cu	30–100	17.5	0.48	76.2	4.2(H_2_O)	85.3 ↓	dehydration
	100–200	7.5	0.21	32.4	1.8(H_2_O)	131.2 ↓	dehydration
	200–900	18.8	0.52	81.9	SO_3_	498.5 ↓ 515.1 ↓ 565.8 ↓	phase transition -- MnSO_4_ melting

**Table 9:** Thermoanalytical parameters obtained and calculated from the DSC thermogram for the mixed KMn/M^II^ Tutton salts.

Crystal	Temperature Range [°C]	Enthalpy [kJ/mol H_2_O]	Total Enthalpy [kJ/mol]	Energy Density [GJ/m^3^]
KMn/Ni	80.7–176.2	79.4	476.5	2.43
KMn/Cu	56.4–129.6	70.6	423.9	2.14

**Table 10:** Compounds and amounts used in solution preparations.

K_2_SO_4_ [g]	MnSO_4_(H_2_O) [g]	M^II^SO_4_(H_2_O)_n_ [g]	Salt Abbreviation
1.3768	1.3521	NiSO_4_(H_2_O)_7_ 2.2468	KMn/Ni
1.3768	1.3521	CuSO_4_(H_2_O)_5_ 1.9974	KMn/Cu

## Data Availability

Crystal structure information is available at www.ccdc.cam.ac.uk (accessed on 1 October 2023) Cambridge Structural Database under the numbers 220517 and 220516.
